# Gait Speed Trajectory During the Six-Minute Walk Test in Multiple Sclerosis: A Measure of Walking Endurance

**DOI:** 10.3389/fneur.2021.698599

**Published:** 2021-07-26

**Authors:** Shanshan Chen, Salvador Sierra, Yongyun Shin, Myla D. Goldman

**Affiliations:** ^1^Department of Biostatistics, Virginia Commonwealth University, Richmond, VA, United States; ^2^Department of Internal Medicine, School of Medicine, Virginia Commonwealth University, Richmond, VA, United States; ^3^Department of Neurology, School of Medicine, Virginia Commonwealth University, Richmond, VA, United States

**Keywords:** six-minute walk test, multiple sclerosis, gait speed trajectory, walking endurance, linear mixed-effects model

## Abstract

**Background:** The six-minute walk (6MW) test is a validated assessment method in Multiple Sclerosis (MS) research. While the total distance covered during six minutes (**6MW^TD^**) is often used as the standard measurement of gait capacity (i.e., the maximum distance one can achieve), we hypothesize that endurance (i.e., ability to maintain speed over a prolonged time) can be inferred by the gait speed trajectory (GST) during the 6MW test (**6MW^GST^**).

**Objective:** To characterize group differences in **6MW^GST^** between MS patients and healthy controls (HCs), and to assess information added by **6MW^GST^** for discerning between MS patients and HCs.

**Methods:** We performed a secondary data analysis on a cross-sectional cohort of 40 MS and 20 HC subjects with three repeated 6MW tests. We modeled **6MW^GST^** using a linear mixed-effects model with time in minutes and replicated walks nested within individuals. We compared the discernibility of **6MW^GST^** with that of conventional metrics using likelihood ratio tests and receiver operating characteristic (ROC) analysis on logistic regression models.

**Results:** MS subjects showed a concave, quadratic GST during 6MW tests, slowing down more than the HC subjects, especially at the beginning of 6MW tests. Despite accelerating at the end of the 6MW, MS subjects were unable to attain or surpass their initial 6MW gait speeds. **6MW^GST^** added useful information (*p* = 0.002) to the conventional metrics (e.g., **6MW^TD^**) for discerning between MS and HC subjects, and increased the area under the ROC curve from 0.83 to 0.93 (*p* = 0.037).

**Conclusions:** The distinctive **6MW^GST^** pattern of MS patients provided increased discernibility compared with currently used gait metrics. Both gait capacity measured by the **6MW^TD^**, and gait endurance measured by parameters of **6MW^GST^**, are significant functional indicators for the MS population.

## Introduction

Gait impairment is a central and ultimately inevitable aspect of multiple sclerosis (MS) that can be expressed as a decrease in gait speed, endurance, and balance. Thus, gait assessment is commonly employed in the evaluation of MS disability. Several timed-walk tests have been used in MS assessment that vary in the distance and duration of walking (e.g., timed 25-foot walk, two- and six-minute walk tests) (1–4). The six-minute walk (6MW) test has been used within MS studies and throughout health science. The 6MW total distance (**6MW^TD^**) is a validated, standard outcome that has demonstrated added value over shorter distance walk tests in that it measures gait capacity (i.e., the ability to achieve maximal distance) (5), offers improved precision (1), correlates with habitual gait performance (6), and correlates with other physiological measures (7). Some researchers have also explored minute-by-minute 6MW data, by computing the percentage change in gait speed in the 6^th^ minute relative to the 1^st^ minute, sometimes referred to as the Δ6MW (2, 3). In the largest study of Δ6MW in MS participants to date, Leone et al. (3) devised the distance walk index (DWI), which is the percentage change in gait speed in the nth minute relative to the 1^st^ minute. Specifically, they used DWI at the 6^th^ minute (i.e., Δ6MW as in other literature) to subgroup MS patients, demonstrating that more than 50% of their MS patients decelerated during 6MW tests. Van Geel et al. assessed the day-to-day reliability of Δ6MW, confirming consistent deceleration patterns in MS patients on two separate days (8). Ramari et al. demonstrated that in females with mild MS, knee flexor strength and balance are linked to the deceleration pattern in the 6MW test (9). Using temporally more continuous data collected by wearable sensors, Shema-Shiratzky et al. also demonstrated significant deterioration in cadence, stride regularity, and gait complexity among MS patients during 6MW tests (10).

Collectively, most literature on the 6MW test has been cross-sectional and based on a single 6MW. Moreover, the existing metric for measuring within-walk performance, Δ6MW, relies on the assumption that change in gait speed is linear, and can thus be captured by the gait speed in the first and last minutes alone. However, it is more likely that changes in gait speed vary across all six minutes, and the gait speed trajectories in the 6MW (**6MW^GST^**) may carry additional information which would more precisely measure *endurance*. We hypothesize that **6MW^GST^** will provide additional information that goes beyond total distance (or equivalently, average gait speed), to give a true representation of endurance. In this manuscript, we analyze data from a previously published cohort (1), who performed three repeated 6MWs in a single day, using linear mixed-effects (LME) models. An LME model allows us to incorporate the full information from the minute-by-minute **6MW^GST^** and assess the effect of MS status on gait endurance (measured by changes in **6MW^GST^**), whilst accounting for idiosyncratic differences in the baseline gait speed.

## Methods

### Participants

A previously published cross-sectional study with a cohort of 60 subjects was analyzed (1). This cohort included 40 MS patients and 20 HC subjects who completed three repeated 6MW tests in a single day (1). Ethical approval for this study was obtained from the Cleveland Clinic Foundation Institutional Review Board (IRB). All participants provided written informed consent prior to any study procedures. The University of Virginia IRB provided approval for secondary analyses of the de-identified data presented herein.

The MS subjects had a diagnosis of clinically definite MS according to the McDonald Criteria (11) and were recruited from outpatients at the Cleveland Clinic neurology department. HC subjects were recruited through MS subjects (e.g., a spouse or a friend). All subjects were aged 18–64 years old, reported being able to walk for six minutes, and had an EDSS (Expanded Disability Status Scale) score no higher than 6.5. Exclusion criteria for all subjects included neurological impairment from other diagnoses, orthopedic limitations, morbid obesity (BMI > 40), or known cardiac or respiratory disease. All fatigue-related medications (e.g., dalfampridine or modafinil) were withheld 48 hours before the study visit to avoid underlying changes in gait function being obscured. Additional details regarding the study population can be found in the primary publication (1).

### Clinical Assessment and Disability Measures

Baseline demographics, medical history, and medications were documented. MS-related disability was assessed using the EDSS (12). EDSS severity was further classified by a certified Neuro-status examiner [MDG]. The MS Functional Composite was also collected for further disability assessment. The MSFC is a composite measure of disability that includes a quantitative test of ambulation (timed 25-foot walk, T25FW) (13).

### Six-Minute Walk (6MW) Tests

All 6MW tests took place in a 175-foot hallway using the protocol developed and validated by Goldman et al. (1). Subjects were asked to *walk as far and as fast as possible* and used their usual assistive devices. Subjects arrived at 9:00 a.m. for 6MW tests to eliminate any time-of-day variability and completed three 6MW tests (i.e., three replicates) during a single visit. To eliminate residual fatigue from the previous walk, subjects rested at least 30 minutes between tests. The minute-by-minute distance was recorded for each walk using floor markers at 8.5-foot intervals. Thus, each subject's 6MW test had one repeated-measure outcome at six time points for gait speed (i.e., distance walked per minute), and three explanatory variables—Time (indexed as 0, 1, 2, 3, 4, 5), Replicate (indexed as 0, 1, 2) as a categorical variable, and the MS status of the subject as a binary indicator (MS =1, HC = 0).

### Statistical Analysis

We performed data analysis in Matlab 2019b (Mathworks Inc., Natick, Massachusetts) and R Studio (R version 3.6.3, RStudio Inc., Boston, Massachusetts). To accommodate the temporal variation in speed, and replicated walks nested within subjects, we fit LME models with the minute-by-minute gait speed data (or distance walked per minute) as the outcome variable. As preliminary analysis of the collected data revealed quadratic trajectories, we fit and tested the subject-specific linear and quadratic effects of time for MS and HC subjects. We also tested these temporal effects moderated by MS status.

LME models allow us to estimate the random variation of the **6MW^GST^** over time, controlling for the effects of MS and other covariates. To demonstrate this advantage, we fit an LME model after dropping all MS status related terms, and estimated parameters of subject-specific **6MW^GST^** (i.e., subject-specific intercepts and linear and quadratic slopes). These subject-specific metrics were then used as the predictors of a logistic regression model with MS status as the outcome variable. We compared the discernibility of the predicted subject-specific **6MW^GST^** with other conventional metrics such as **6MW^TD^**, Δ6*MW*, and T25FW. The Δ6*MW* (3, 14) is calculated as:

$$\begin{array}{l} \Delta 6MW=\;\frac{\;D_{6}-D_{1}}{D_{1}}\times 100\% \end{array}$$

*D*_6_ is the gait speed (or distance covered) in the 6^th^ minute and *D*_1_ is the gait speed in the 1^st^ minute. For comparison, we used the MS status as a binary outcome, and developed five nested logistic regression models: (1) Model A contains parameters of subject-specific **6MW^GST^** (i.e., subject-specific intercepts and linear and quadratic slopes estimated from an LME model) and the **6MW^TD^**; (2) Model B uses Δ6*MW* and **6MW^TD^** (3) Model C uses **6MW^TD^**; (4) Model D uses the T25FW test (short-distance timed walk test); (5) Model E uses two covariates, age and sex, and is the baseline model in our study. Models A, B, C, and D were all adjusted for age and sex. For each model, we obtained the area under the receiver operating characteristic curve (AUROC) and the likelihood ratio χ^2^ statistic (tested against an intercept-only null model). We tested added information by comparing AUROC using DeLong's tests and likelihood ratio tests across pairs of models (15).

## Results

60 subjects (40 MS and 20 HC) participated in the study. Baseline characteristics for both study populations are presented in Table 1. In our MS sample, there were 3.4 times as many females as males. This is consistent with the prevalence of MS, i.e., females are three times more likely to develop MS than males. HC subjects were predominantly male, since we actively recruited spouses of MS patients as HC subjects. For the MS sample, the median EDSS was 3 with a range of 0–6.5. Apart from sex and height, there were no significant differences in baseline demographic characteristics between MS and HC participants [age (*p* = 0.461), height (*p* = 0.038), weight (*p* = 0.225) and BMI (*p* = 0.882)]. There were, however, significant differences in gait function. MS subjects covered less distance in the first minute, the last minute, and across the 6 minutes, than HC subjects [Gait speed in 1^st^ min (*p* < 0.001), Gait speed in 6^th^ min (*p* < 0.001), Total distance (*p* < 0.001)].

**Table 1:** Summary statistics of the cross-sectional cohort.

	**HC (N = 20)**	**MS (N = 40)**	**Overall (N = 60)**
**Age (years)**			
Mean (SD)	40.1 (10.5)	42.1 (8.12)	41.4 (8.94)
Median [Min, Max]	45.0 [23.0, 58.0]	44.5 [25.0, 54.0]	45.0 [23.0, 58.0]
**Gender**			
Male	11 (55.0%)	9 (22.5%)	20 (33.3%)
Female	9 (45.0%)	31 (77.5%)	40 (66.7%)
**Height (inches)**			
Mean (SD)	68.1 (4.11)	65.7 (4.00)	66.5 (4.15)
Median [Min, Max]	67.0 [62.0, 76.0]	65.3 [56.0, 76.0]	66.0 [56.0, 76.0]
**Weight (lbs)**			
Mean (SD)	183 (41.7)	169 (40.9)	174 (41.3)
Median [Min, Max]	187 [119, 250]	158 [114, 300]	170 [114, 300]
**BMI (kg/m^2^** **)**			
Mean (SD)	28.6 (4.81)	28.4 (5.06)	28.5 (4.94)
Median [Min, Max]	28.3 [20.9, 37.2]	27.2 [19.0, 38.6]	27.5 [19.0, 38.6]
**Gait Speed in 1^st^** **Min (feet/minute)**			
Mean (SD)	339 (27.4)	293 (59.9)	308 (55.7)
Median [Min, Max]	336 [300, 394]	300 [173, 425]	313 [173, 425]
**Gait Speed in 6^th^** **Min (feet/minute)**			
Mean (SD)	339 (30.4)	279 (63.1)	299 (61.2)
Median [Min, Max]	339 [297, 406]	294 [139, 377]	308 [139, 406]
**6MW Total Distance (6MW^TD^** **)**			
Mean (SD)	2010 (165)	1680 (352)	1790 (338)
Median [Min, Max]	2020 [1780, 2340]	1750 [944, 2210]	1830 [944, 2340]
**Δ6MW (%)**			
Mean (SD)	0.14 (3.54)	−4.79 (6.86)	−3.15 (6.38)
Median [Min, Max]	0.69 [-6.99, 6.55]	−4.21 [-25.4, 8.51]	−1.99 [-25.4, 8.51]
**Timed 25 foot-walk (T25FW) (seconds)**			
Mean (SD)	4.27 (0.477)	5.38 (1.67)	5.01 (1.48)
Median [Min, Max]	4.25 [3.35, 5.45]	4.78 [3.40, 10.3]	4.50 [3.35, 10.3]

Results from the LME model are listed in Table 2. To obtain the nadir times (here 2.7 for the HC, and 4.13 for the MS patients) of the estimated quadratic curves, we rearranged the quadratic time effects by completing the squares:

$$\begin{array}{r} HC:{\left(1.11\times Time-2.70\right)}^{2}-\;7.3\\ MS:{\left(1.26\times Time-4.13\right)}^{2}-\;17.0 \end{array}$$

We first adjusted for age, sex, height, and weight, and then removed these variables from the final model as their effects are insignificant. On average, MS subjects walked 42.73 feet/minute slower than the HCs at the baseline (*p* = 0.003). After the baseline, using the trajectory −42.73−4.45 × Time+.37 × Time^2^, MS patients continued to walk 46.81, 50.15, 52.75, 54.61, 55.73 feet/minute slower than the HCs in the subsequent minutes. Both MS and HC subjects decelerated during the first half of the 6MW, but the MS patients decelerated by 4.5, 3.5, 3.0 feet/minute^2^ more severely in the 2^nd^ to the 4^th^ minutes. In the second half of the 6MW, the HC subjects began to accelerate following a nadir in the 3^rd^ minute. In contrast, MS participants were unable to accelerate as early as HCs. As a result, at the end of the 6MW, HC subjects were able to walk as fast as at the baseline, whereas MS subjects ended up far below their baseline speed. Over the repeated walks, the HC group increased gait speed during the second walk (by 9.9 feet/minute, *p* = 0.003) and the third walk (by 7.5 feet/minute, *p* = 0.021), whereas the MS group only slightly increased gait speed over the repeated walks.

**Table 2:** Results from the linear mixed-effects model.

	**Outcome: Gait Speed during 6MW test**		
**Fixed effects**	**Estimates**	**95% CI**	***p***
(Intercept)	332.94	311.31 – 354.56	<0.001
MS	−42.73	−69.74 – −15.71	0.003
Time (0-5)	−5.98	−8.42 – −3.55	<0.001
Time^2^	1.23	0.82 – 1.64	<0.001
2^nd^ Replicate	9.89	3.56 – 16.22	0.003
3^rd^ Replicate	7.49	1.16 – 13.83	0.021
MS * Time	−4.45	−7.44 – −1.47	0.004
MS * Time^2^	0.37	−0.13 – 0.87	0.147
MS * 2^nd^ Replicate	−7.77	−15.53 – −0.02	0.051
MS * 3^nd^ Replicate	−3.78	−11.54 – 3.98	0.339
**Random effects**	**Standard deviation**	**Correlation coefficient**	
Intercept—replicate	12.91	−0.91	
Time—replicate	1.26		
Intercept—subject	47.73	0.20	
Time—subject	2.59		
Residual variance	97.11		

“Intercept” reveals the gait speed at baseline (in our case the gait speed in the 1^st^ minute of the 1^st^ 6MW replicate) of the HC subjects. “Time” captures the linear temporal change in gait speed and “Time^2^″ captures the quadratic temporal change in gait speed of HC subjects. “2^nd^ Replicate” and “3^rd^ Replicate” capture the deviation in gait speed of the second and the third replicates, respectively, from the first walk among HCs. The “MS” main effect captures the deviation in baseline gait speed of the MS patients relative to HC subjects. The interaction effects, “MS ^*^ Time”, “MS ^*^ Time^2^”, “MS ^*^ 2^nd^ Replicate”, and “MS ^*^ 3^rd^ Replicate” capture the differences in time effects or replicated walk effects of MS patients relative to HCs. Our main interest is to assess how different the MS effects are compared to those of HCs.

**Figure 1:**
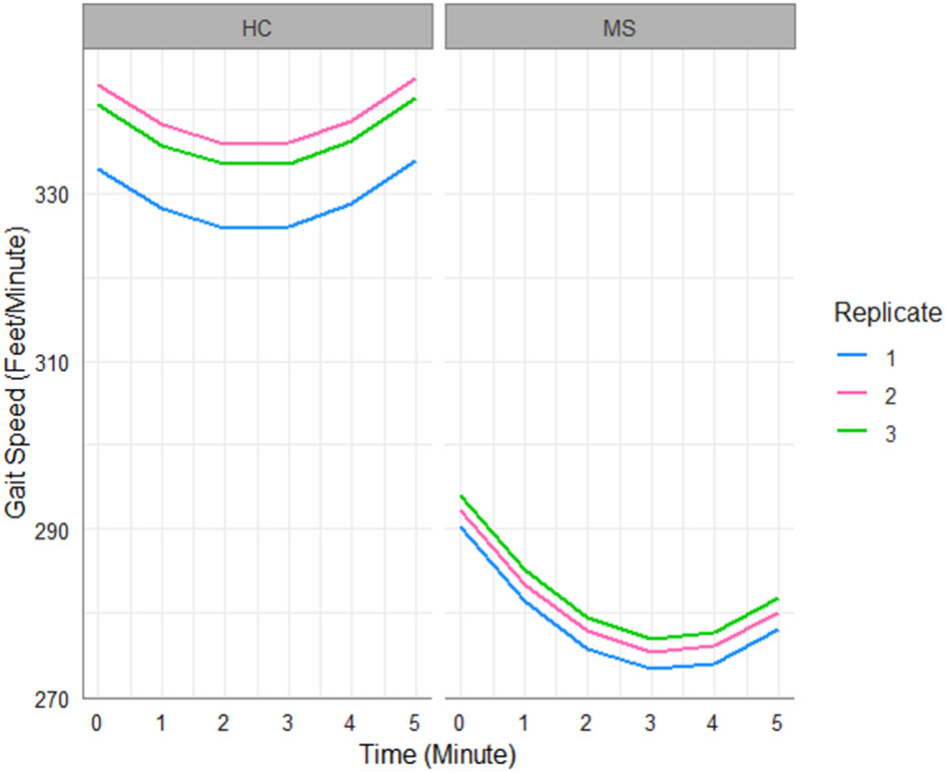
Gait speed trajectories predicted by the fitted LME model in Table 2.

Figure 1 shows the predicted trajectories of MS patients and HCs over replicated 6MW tests, as an illustration of our findings detailed in Table 2. First, there is a large difference in gait speed between the groups. HC subjects, despite decelerating in the first half of the 6MW test, exhibit acceleration over the second half of the 6MW test. In contrast, MS participants decelerate more rapidly in the first half of the 6MW and are unable to return to their baseline speed in the final minute. Moreover, while the HCs are able to improve their speeds over the replicated walks, the MS patients show no significant change in their gait speed over repeated walks. These findings suggest such differences are more likely to reflect the inherent pathology among MS subjects.

**Figure 2:**
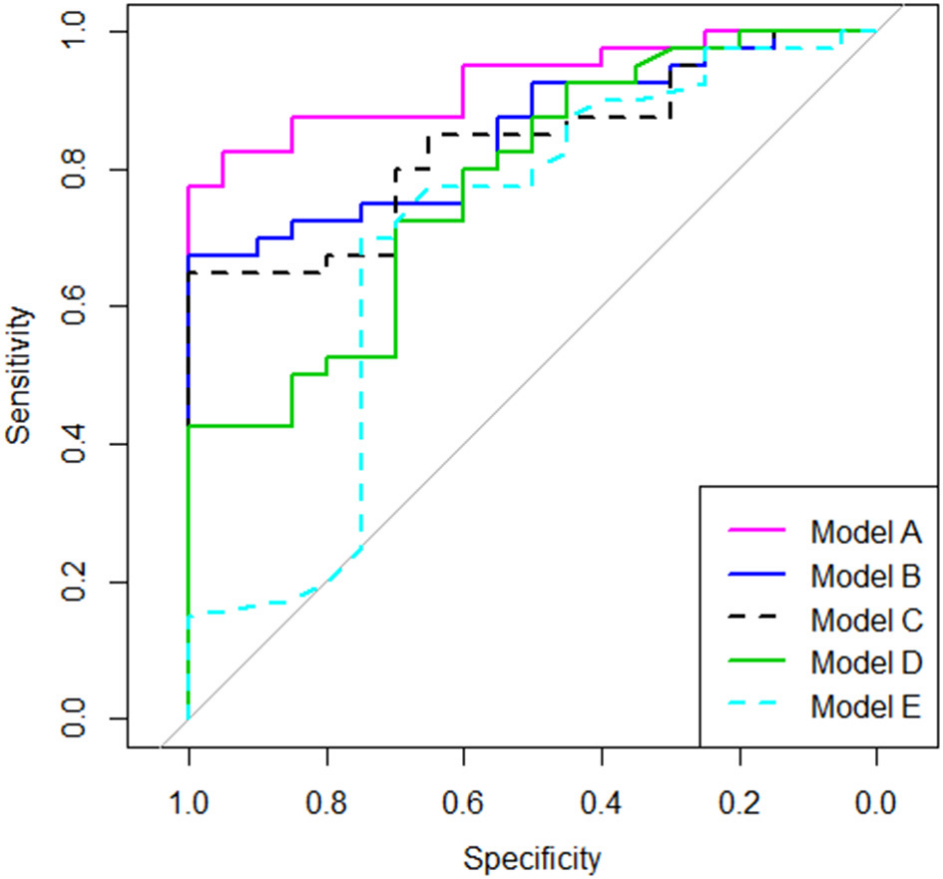
Comparing discriminative information using ROC curves.

Using nested logistic regression models, we compared the conventional metrics such as **6MW^TD^**, Δ6MW, and T25FW with the **6MW^GST^** parameters estimated by the LME model as explained in the Statistical Analysis section. Table 3 shows model comparison results. Our Model A (**6MW^GST^** + **6MW^TD^**) added significantly more information (*p* = 0.002) to Model C (**6MW^TD^**) and improved predictability (*p* = 0.037), whereas Model B (Δ6*MW* + **6MW^TD^**) did not. While Model D (Timed 25-foot walk test) added more information to the baseline model (Model E), it had the lowest AUROC among Models A-D. Overall, Model A with the proposed parameters of the **6MW^GST^** provided the most information about MS disease status among the models compared, whereas Δ6*MW* was no better than **6MW^TD^**. The ROC curves are shown in Figure 2.

**Table 3:** Discriminative information comparison using logistic regression models with MS status as the outcome.

	**Metrics of model** **discriminability**		**Model comparison**		
**Models** **(Predictors)**	**AUROC (95% CI)**	**Likelihood** **ratio χ^2^**	**Comparison pair**	***p*** **-value by Delong's** **test**	***p*** **-value by likelihood** **ratio test**
A: (**6MW^GST^** + **6MW^TD^** + age + sex)	0.93 (0.86–0.99)	38.3	Model A against Model C	0.037	0.002
B: (Δ6MW + **6MW^TD^** + age + sex)	0.85 (0.76–0.94)	25.8	Model B against Model C	0.491	0.109
C: (**6MW^TD^** + age +sex)	0.83 (0.73–0.93)	23.2	Model C against Model E	0.058	<0.001
D: (T25FW + age + sex)	0.78 (0.65–0.90)	15.6	Model D against Model E	0.129	0.006
E: (age + sex)	0.70 (0.54–0.86)	8.2	Model A against Model B	0.045	N/A

## Discussion

In this study, we demonstrate consistent and significant deceleration patterns, an indicator of diminished endurance, among MS patients during repeated 6MW tests. Our finding underscores that the less-investigated aspect of the 6MW tests—the temporal change in minute-by-minute gait speed—offers a unique characterization of gait beyond **6MW^TD^**. By testing the group differences in gait speed trajectories between MS and HC subjects, our LME model confirms a deterioration in both gait capacity and endurance among MS subjects. The LME model takes into consideration sources of variation in the gait speed outcome such as the subject-specific baseline gait speed and repeated walks, thus better elicits gait degradation due to MS-disability status. Using this model, we identified no significant change in **6MW^GST^** over repeated 6MW tests among MS patients, indicating that a single 6MW test may be adequate to assess gait endurance for MS patients. With the MS status as a binary outcome, we also compared logistic regression models using parameters of **6MW^GST^** estimated by an LME model against models using other conventional metrics (i.e., Δ6*MW*, **6MW^TD^**, and the T25FW metric). Ultimately, our analysis showed that the subject-specific parameters of **6MW^GST^** capture additional information about gait endurance in the MS population, beyond the gait capacity measured by total distance.

MS patients are recognized to have denuded axons due to demyelination. The consequence of this is impaired nerve conduction. Fampridine, a potassium channel blocker, has been demonstrated to improve gait speed with a presumed mechanism of action of sustained conduction across demyelinated axons secondary to increased potassium availability (16). We posit that the differences in the subject-specific temporal variation in gait speed may be an indicator of this recognized conduction failure in MS individuals. Specifically, a deceleration pattern in the **6MW^GST^** may be due to conduction failure among those MS patients with denuded axons. Were this to be the case, **6MW^GST^** could serve as a potential biomarker when screening subjects for future remyelination trials, enrolling only decelerators as a recruitment enrichment strategy. The underlying pathophysiology driving 6MW deceleration in MS warrants further investigation which is not explored in this manuscript.

Although other studies found a strong correlation between gait speeds measured in 6MW and 2MW tests, the latter can only capture gait capacity (17, 18), and are unable to assess gait endurance. Similarly, the 500-meter walk of the EDSS, and the T25FW test provide only a single endpoint for gait capacity evaluation, omitting the rich within-subject temporal patterns during walking. Given that the time needed to complete the 6MW (i.e., six minutes) is not substantially greater than the time needed for the 500-meter walk of the EDSS, and that the 6MW test can provide critical information about endurance and gait capacity at the same time, the 6MW test could be integrated into the EDSS as a substitute for the 500-meter walk.

One limitation of this work is the recruitment of patients' spouses as HC subjects. As MS is three times more prevalent in females (19), our control subjects tended to be male. Although we did not find any gender or height effect on the outcome, a potential gender confounding effect may exist in our data (e.g., females tend to walk slower than males). It is worth noting that while this confounding factor may impact **6MW^TD^**, by focusing on the within-subject pattern, our proposed **6MW^GST^** is robust to height or baseline gait speed differences. Also, although our sample size is sufficient to carry out the LME modeling, it is too small for developing diagnostic prediction models that could validate **6MW^GST^** metrics. Lastly, we cannot track whether these **6MW^GST^** metrics worsen longitudinally as these data were collected on a single day. Additional studies with larger sample sizes will be required to further characterize the longitudinal changes in **6MW^GST^** among MS patients.

In conclusion, **6MW^GST^** is a promising measure for assessing impaired gait function that merits further investigation. Our results demonstrate that the **6MW^GST^** quantified by LME models is more informative than other gait outcome measures. Further studies will be necessary to confirm and expand our understanding of the potential underlying pathobiological relationship between the deceleration slope and associated measures of axonal integrity and myelination.

## Data Availability Statement

The original contributions presented in the study are included in the article/supplementary material, further inquiries can be directed to the corresponding author.

## Ethics Statement

The studies involving human participants were reviewed and approved by Cleveland Clinic Foundation IRB Committee. The patients/participants provided their written informed consent to participate in this study.

## Author Contributions

MG: data acquisition. SC and MG: study concept, analysis, and design. YS: analysis and results interpretation. All authors drafting the manuscript and figures.

## Conflict of Interest

The authors declare that the research was conducted in the absence of any commercial or financial relationships that could be construed as a potential conflict of interest.

## Publisher's Note

All claims expressed in this article are solely those of the authors and do not necessarily represent those of their affiliated organizations, or those of the publisher, the editors and the reviewers. Any product that may be evaluated in this article, or claim that may be made by its manufacturer, is not guaranteed or endorsed by the publisher.
